# The Effect of Steaming and Fermentation on Nutritive Values, Antioxidant Activities, and Inhibitory Properties of Tea Leaves

**DOI:** 10.3390/foods10010117

**Published:** 2021-01-08

**Authors:** Chaowanee Chupeerach, Amornrat Aursalung, Thareerat Watcharachaisoponsiri, Kanyawee Whanmek, Parunya Thiyajai, Kachakot Yosphan, Varittha Sritalahareuthai, Yuraporn Sahasakul, Chalat Santivarangkna, Uthaiwan Suttisansanee

**Affiliations:** 1 Institute of Nutrition, Mahidol University, Salaya, Phuttamonthon, Nakhon Pathom 73170, Thailand; chaowanee.chu@mahidol.ac.th (C.C.); amornrat.aur@mahidol.ac.th (A.A.); thareerat.wat@hotmail.com (T.W.); kanyaweebiosci@gmail.com (K.W.); parunya.thy@mahidol.ac.th (P.T.); kachakot.yp@gmail.com (K.Y.); varittha.sri@hotmail.com (V.S.); yuraporn.sah@mahidol.ac.th (Y.S.); chalat.san@mahidol.ac.th (C.S.); 2 Food and Nutrition Academic and Research Cluster, Institute of Nutrition, Mahidol University, Salaya, Phuttamonthon, Nakhon Pathom 73170, Thailand

**Keywords:** Cha-miang, fermented tea, bioactive compounds, nutritive values, enzyme inhibitory activities

## Abstract

Fermented tea (Cha-miang in Thai) is a local product made by traditional food preservation processes in Northern Thailand that involve steaming fresh tea leaves followed by fermenting in the dark. Information on changes in nutritive values, bioactive compounds, antioxidant activities, and health properties that occur during the steaming and fermenting processes of tea leaves is, however, limited. Changes in nutritive values, phenolics, antioxidant activities, and in vitro health properties through inhibition of key enzymes that control obesity (lipase), diabetes (α-amylase and α-glucosidase), hypertension (angiotensin-converting enzyme (ACE)), and Alzheimer’s disease (cholinesterases (ChEs) and β-secretase (BACE-1)) of fermented tea were compared to the corresponding fresh and steamed tea leaves. Results showed that energy, carbohydrate, and vitamin B1 increased after steaming, while most nutrients including protein, dietary fiber, vitamins (B2, B3, and C), and minerals (Na, K, Ca, Mg, Fe, and Zn) decreased after the steaming process. After fermentation, energy, fat, sodium, potassium, and iron contents increased, while calcium and vitamins (B1, B2, B3, and C) decreased compared to steamed tea leaves. However, the contents of vitamin B1 and iron were insignificantly different between fresh and fermented tea leaves. Five flavonoids (quercetin, kaempferol, cyanidin, myricetin, and apigenin) and three phenolic acids (gallic acid, caffeic acid, and *p*-coumaric acid) were identified in the tea samples. Total phenolic content (TPC) and antioxidant activities increased significantly after steaming and fermentation, suggesting structural changes in bioactive compounds during these processes. Steamed tea exhibited high inhibition against lipase, α-amylase, and α-glucosidase, while fermented tea possessed high anti-ChE and anti-ACE activities. Fresh tea exhibited high BACE-1 inhibitory activity. Results suggest that tea preparations (steaming and fermentation) play a significant role in the amounts of nutrients and bioactive compounds, which, in turn, affect the in vitro health properties. Knowledge gained from this research will support future investigations on in vivo health properties of fermented tea, as well as promote future food development of fermented tea as a healthy food.

## 1. Introduction

Tea (*Camellia sinensis*) is a globally popular drink with a higher consumption rate than coffee, wine, beer, and soft drinks [1]. Tea is also used in both pharmaceutical and industrial applications. The key chemical compounds in tea that control the flavor and taste consist of volatile terpenes, organic acids, caffeine, and polyphenols (present predominantly as catechins; others are gallic acid, caffeic acid, chlorogenic acid, quercetin, kaempferol, and myricetin) [1,2]. Tea has health benefits, with positive effects in terms of antihypertensive, antiarteriosclerotic, hypocholesterolemic, and hypolipidemic activities [3]. Fermented tea is also popular among tea drinkers as a new flavor with diverse bioactive compounds. Thai fermented tea (or Cha-miang) is a local product in Northern Thailand, which serves as an alternative application to consume tea [4]. Conventional methods of fermentation vary among regions; however, the basic procedures include steaming for 1–2 h before fermenting in the dark for several days or up to a year [2]. Instead of drinking tea, the final product of Cha-miang is chewing or eating tea with a sour–bitter taste, consumed as a snack alone or served with salt and other ingredients such as roasted coconut, shredded ginger, and garlic [2]. Previously, chewing Cha-miang was only popular in the elderly. However, chewing has become a normal habit for all ages. Therefore, it is of great interest to investigate the nutrients and health properties of this historical food product for the future development of other functional foods and drinks from Cha-miang.

Cha-miang possesses many beneficial attributes in food, pharmaceutical, and nutraceutical applications. It is rich in phenolic compounds, with several health benefits including antimicrobial and antioxidant activities [2,5] due to the nutritional biotransformation which occurs during the fermenting process [6]. Cha-miang is considered to be a unique cultural food with limited consumption among locals; nevertheless, beneficial health properties resulting from variations in bioactive compounds and nutritional changes have not been previously investigated. Currently, regulations on the activity of key enzymes that can ameliorate the incidence of noncommunicable diseases (NCDs) such as obesity, diabetes, Alzheimer’s disease (AD), and hypertension have received attention due to enzyme characteristics in enzyme–substrate specificity [7]. Obesity is a major global public health problem that is continuously increasing due to lifestyle changes. Obesity can be controlled through the triglyceride-hydrolyzing enzyme, lipase. Inhibition of lipase decreases the rate of fat digestion and, thus, absorption [8]. Likewise, a new approach for diabetic management involves inhibition of the carbohydrate-hydrolyzing enzymes, α-amylase and α-glucosidase [9,10], while prevention of hypertension is associated with control of the angiotensin-converting enzyme (ACE) [11]. Recent pathways to control AD include termination of the physiological role of cholinergic synapses and β-amyloid formation via inhibition of the key enzymes, acetylcholinesterase (AChE), butуrylсholinesterase (BChE), and β-secretase (β-site amyloid precursor protein cleaving enzyme 1 or BACE-1) [12,13,14,15]. However, despite this information, limited data exist regarding the effects of steaming and fermenting processes on nutritive values, bioactive compounds, antioxidant activities, and prevention of obesity, diabetes, hypertension, and AD via inhibition of the key enzymes controlling these diseases. Therefore, here, changes in nutritive values, bioactive compounds, and antioxidant activities during Cha-miang preparation (fresh tea leaves compared to steamed and fermented tea leaves) were investigated. Changes in in vitro health properties through inhibition of the key enzymes that control obesity (lipase), diabetes (α-glucosidase and α-amylase), hypertension (ACE), and AD (AChE, BChE, and BACE-1) of fresh tea leaves compared to the corresponding steamed and fermented tea leaves were also examined. Knowledge gained from these experiments can be used to support the consumption of Cha-miang as a healthy food and promote the future development of other functional foods from Cha-miang.

## 2. Materials and Methods

### 2.1. Sample Preparation, Microbiological Analysis, and Extraction

Fresh tea leaves (*Camellia sinensis* var. assamica), as well as steamed and fermented teas, were collected from Chiang Rai Province, Thailand in October 2017. Briefly, fermented tea was prepared by collecting the tea leaves into small bundles using bamboo strips, followed by steaming at 100 °C for 1 h before cooling. The steamed tea leaves underwent nonfilamentous fungal-based fermentation (or a single-step fermentation process) [2]. The steamed tea leaves were packed in a cement container and tightly wrapped in the dark at ambient temperature to minimize air content and to initiate the fermentation in an anaerobic environment. Fermentation occurred for 1 month. The fresh, steamed, and fermented tea leaves (1 kg each, *n* = 3) were collected throughout the preparation to ensure homogeneity of the leaves.

To perform the microbiological analysis, 10 g of fermented tea leaves were aseptically weighed and homogenized with 90 mL of 0.85% (*w*/*v*) NaCl solution. The mixture was subjected to 10-fold serial dilution and analyzed using the spread plate technique. Media used for microbial enumeration included plate count agar (PCA) (Merck, Darmstadt, Germany) incubated at 37 °C for 48 h for total viable bacteria, de Man Rogosa and Sharpe (MRS) agar (Difco, Franklin Lakes, NJ, USA) incubated at 37 °C for 48 h for total lactic acid bacteria, and yeast mold (YM) agar (Difco, Franklin Lakes, NJ, USA) incubated at 25 °C for 3–5 days for yeast and mold. Microbial numbers were calculated and expressed as log colony-forming units (CFU) per gram of sample.

The samples were prepared for antioxidant analysis and in vitro health properties by freeze-drying for 3 days in a freeze-dryer (Heto PowerDry PL9000 series from Heto Lab Equipment, Allerod, Denmark). Dry samples were then ground into fine powder using a grinder (Philips 600W series from Philips Electronics Co., Ltd., Jakarta, Indonesia) before packing in vacuum aluminum foil bags and keeping at −20 °C in a freezer until required for further analysis. Moisture content was analyzed utilizing a Halogen moisture analyzer (HE53 series, Mettler-Toledo AG, Greifensee, Switzerland).

Extractions to determine total phenolic contents, antioxidant activities, and enzyme inhibitory activities were performed as follows: the dry samples (400 mg) were dissolved in 10 mL of distilled water and incubated at 70 °C for 1 h using a water-bath shaker (WNE45 series from Memmert GmBh, Eagle, WI, USA). The supernatant was collected via centrifugation at 3800× *g* (a Hettich^®^ Rotina 38R refrigerated centrifuge from Andreas Hettich GmbH, Tuttlingen, Germany) for 15 min and filtered through a 0.45 µM polyethersulfone (PES) membrane syringe filter. All extracted samples were stored at −20 °C until required for further analysis.

### 2.2. Determination of Nutritive Values

The nutritive values of fresh samples were determined utilizing the standard protocols of the Association of Official Analytical Chemists (AOAC) [16] at the Institute of Nutrition, Mahidol University (ISO/IEC 17025:2005). Nutritional values including moisture content, energy, protein, fat, carbohydrate, total dietary fiber, soluble dietary fiber, insoluble dietary fiber, ash, minerals (Na, K, Ca, Mg, Fe, and Zn), and vitamins (vitamin C, B1, B2, and B3) were reported as per 100 g fresh weight, as shown in Table S1 (Supplementary Materials). To accurately determine the effect of the steaming and fermenting processes, nutritional contents were calculated and reported per 100 g dry weight.

Moisture content was determined by drying the fresh sample in a 100 °C hot-air oven (Memmert Model UNE 500, Eagle, WI, USA) until the sample weight was constant (AOAC 930.04, 934.01).

Protein content was determined via the Kjeldahl method using digestion and distillation units (Buchi Model K-435 and B-324, Flawil, Switzerland, respectively) and then calculated using a conversion factor of 6.25 (AOAC 992.23).

Crude fat content was analyzed via acidic digestion and extracted with petroleum ether using a Soxtec System (Tecator Model 1043, Hoganas, Sweden) (AOAC 948.15, 945.16).

Total dietary fiber, soluble dietary fiber, and insoluble dietary fiber were determined via the enzymatic gravimetric method (AOAC 991.42 for soluble dietary fiber and AOAC 991.43 for insoluble dietary fiber). The sample was sequentially digested with α-amylase, amyloglucosidase, and protease. Soluble and insoluble dietary fibers were calculated separately, while total dietary fiber was indirectly calculated as the sum of both dietary fibers.

Ash content was determined via incineration in a muffle furnace (Carbolite Model CWF 1100, Hope, UK) at 550 °C (AOAC 930.30, 945.46).

Carbohydrate and energy were derived via calculation using the following equations:

Total carbohydrate (g) = 100 − moisture − protein − total fat − ash.

Energy (kcal) = (total carbohydrate × 4) + (protein × 4) + (total fat × 9).

Ash residue was used to determine calcium, sodium, and potassium contents using a flame atomic absorption spectrophotometer (AAS, Thermo S series, Waltham, MA, USA) (AOAC 985.35), while magnesium, iron, and zinc contents were analyzed using inductively coupled plasma optical emission spectroscopy (ICP-OES) (AOAC 984.27).

Vitamin C was determined by extracting the samples in 10% (*v*/*v*) metaphosphoric acid (MPA) before filtering through a 0.22 µM polytetrafluorothylene (PTFE) membrane syringe filter and loading to a high-performance liquid chromatography (HPLC) system utilizing a Waters 515 pump (Waters Corporation, Milford, MA, USA), a Zorbax original ODS column (5 μm, 250 × 4.6 × 10^−3^ m, Agilent Technologies, Santa Clara, CA, USA), and an isocratic solvent system (0.5% (*v*/*v*) KH_2_PO_4_, adjusted to pH 2.5 with H_3_PO_4_) with a flow rate of 0.8 mL/min [17]. Vitamin C was visualized at 254 nm using an ultraviolet/visible light (UV/Vis)-975 detector (JASCO International Co., Ltd., Tokyo, Japan).

Vitamin B1 (thiamine) and B2 (riboflavin) were extracted following the protocol of AOAC method no. 942.23 and 970.65, respectively [16], and detected utilizing an HPLC system with a pump (LC-20AT pump, Shimadzu Scientific Instrument, Columbia, MD, USA), and fluorescence detector (FP-920, JASCO International Co., Ltd., Tokyo, Japan). Vitamin B1 and B2 were separated by a Luna^®^ C18(2) 100A column (5 μm, 250 × 4.6 × 10^−3^ m, Phenomenex, Torrance, CA, USA) under an isocratic solvent system (50% (*v*/*v*) methanol) with a flow rate of 1.0 mL/min [18,19].

Vitamin B3 (niacin) was extracted following the protocol of AOAC method no. 961.14 [16] and detected utilizing an HPLC system with a pump (1200 series G1310A isocratic pump, Agilent Technologies, Santa Clara, CA, USA), and variable wavelength detector (VWD) UV detector (1100 series G1314B, Agilent Technologies, Santa Clara, CA, USA). Vitamin B3 was separated by a Luna^®^ C8(2) 100A column (5 μm, 250 × 4.6 × 10^−3^ m, Phenomenex, Torrance, CA, USA) under an isocratic solvent system (15% (*v*/*v*) methanol) with a flow rate of 1.0 mL/min [20,21].

### 2.3. Determination of Phenolic Profiles

Phenolic profiles were determined utilizing the Agilent 1100 HPLC system with a photodiode array detector (Agilent Technologies, Santa Clara, CA, USA), a 5 µm Zorbax Eclipse XDB-C_18_ column (150 × 4.6 mm from Agilent Technologies), and a solvent system, as described previously [22]. Phenolics were identified at 280 nm and 325 nm using a ChemStation software (Agilent Technologies, Santa Clara, CA, USA) by comparing the retention time (*t_R_*) and spectral fingerprint with standards. The standards for phenolic acids were caffeic acid (>98.0% HPLC, T), chlorogenic acid (>98.0% HPLC, T), *p*-coumaric acid (>98.0% GC, T), ferulic acid (>98.0% GC, T), 4-hydroxybenzoic acid (>99.0% GC, T), sinapic acid (>99.0% GC, T), and syringic acid (>97.0% T) from Tokyo Chemical Industry (Tokyo, Japan), and gallic acid (97.5–102.5% T) from Sigma-Aldrich (St. Louis, MO, USA). Standards for flavonoids were apigenin (>98.0% HPLC), kaempferol (>97.0% HPLC), hesperidin (>90.0% HPLC, T), myricetin (>97.0% HPLC), luteolin (>98.0% HPLC), quercetin (>98.0% HPLC, E), and naringenin (>93.0% HPLC, T) from Tokyo Chemical Industry (Tokyo, Japan), and isorhamnetin (≥99.0% HPLC) from Extrasynthese (Genay, France). Anthocyanins were visualized at 524 nm, in which the *t_R_* and spectral fingerprint were compared with standards including cyanidin (≥96.0% HPLC), malvidin (≥97.0% HPLC), delphinidin (≥97.0% HPLC), petunidin (≥95.0% HPLC), and peonidin (≥97.0% HPLC) from Extrasynthese (Genay, France). The validation parameters for HPLC analysis are shown in Table S2 (Supplementary Materials) and HPLC chromatograms are shown in Figure S1 (Supplementary Materials).

Total phenolic content (TPC) was analyzed utilizing Folin–Ciocalteu’s phenol as a reagent and gallic acid (0–200 µg/mL) as a standard, as previously reported [23,24]. Results were monitored at 765 nm using a Synergy^TM^ HT 96-well UV/visible microplate reader (BioTek Instruments, Inc., Winooski, VT, USA) and Gen 5 data analysis software.

### 2.4. Determination of Antioxidant Activities

Antioxidant activities were analyzed via 2,2-diphenyl-1-picrylhydrazyl (DPPH) radical scavenging, ferric ion reducing antioxidant power (FRAP), and oxygen radical absorbance capacity (ORAC) assays using well-established protocols [25,26,27,28]. The DPPH radical scavenging assay utilizing DPPH in 95% (*v*/*v*) aqueous ethanol and the Trolox standard (0.01–0.64 mM) was performed as previously described [25,28]. The FRAP assay utilizing FRAP reagent and the Trolox standard (7.81–250.00 µM) was determined according to the previously reported procedures [26,28]. The ORAC assay utilizing fluorescein reagent and the Trolox standard (3.12–100.00 µM) was analyzed as previously described [27,28].

### 2.5. Determination of Enzyme Inhibitory Activities

Lipase inhibitory activity was determined using a well-established protocol as previously described [29]. Briefly, the reaction consisted of 100 µL of 0.01 mg/mL *Candida rugosa* lipase (type VII, ≥700 unit/mg) in 50 mM Tris (pH 8.0) containing 0.1% (*w*/*v*) bovine serum albumin (BSA), 50 µL of 0.2 mM 5-5′-dithiobis(2-nitrobenzoic-*N*-phenacyl-4,5-dimethyуhiаzolium bromide) (DMPTB) in 50 mM Tris (pH 7.2) containing 10 mМ KCl and 1 mM ethylenediaminetetraacetic acid (EDTA), 10 µL of 16 mM 5,5′-dithiobis(2-nitrobenzoic acid) (DTNB) in 50 mM Tris (pH 7.2) containing 10% (*v*/*v*) Triton X-100, 10 mM KC1,and 1 mM EDTA, and 40 µL of extract. Lipase inhibitory activity was monitored at 412 nm using the microplate reader. Results were calculated as percentage inhibition using the following equation:

$$\%\;\mathrm{inhibition}\;=\;\left(1-\;\;\frac{B-b}{A-a}\right)\;\times \;100,$$

where *A* is the initial velocity of the control reaction with enzyme (control), *a* is the initial velocity of the control reaction without enzyme (control blank), *B* is the initial velocity of the enzyme reaction with extract (sample), and *b* is the initial velocity of the reaction with extract but without enzyme (sample blank).

The α-glucosidase inhibitory activity was determined using a well-established protocol as previously described [22]. Briefly, the reaction consisted of 50 µL of 2 mM *p*-nitrophenyl-α-d-glucopyranoside in a 50 mM phosphate buffer (pH 7.0), 100 µL of 0.1 U/mL *Saccharomyces cerevisiae* α-glucosidase (type 1, ≥10 U/mg protein), and 50 µL of extract. The α-glucosidase inhibitory activity was monitored at 405 nm using the microplate reader, and inhibition percentage was calculated as described above.

The α-amylase inhibitory activity was determined using a well-established protocol as previously described [22]. Briefly, the reaction consisted of 50 µL of 30 mM *p*-nitrophenyl-α-d-maltohexaoside in a 50 mM phosphate buffer (pH 7.0) containing 200 mM KCl, 100 µL of 30 mg/mL of porcine pancreatic α-amylase (tуре VII, ≥10 unit/mg), and 50 µL of extract. The α-amylase inhibitory activity was monitored at 405 nm using the microplate reader, and inhibition percentage was calculated as described above.

Acetylcholinesterase (AChE) inhibitory activities were determined using a well-established protocol as previously described [22]. Briefly, the reaction consisted of 100 μL of 20 ng of *Electrophorus electricus* AChE (1000 units/mg) in 50 mM potassium phosphate buffer (KPB) (pH 7.0), 10 μL of 16 mM 5,5-dithio-bis-(2-nitrobenzoic acid) (DTNB), 40 μL of 0.8 mM acetylthiocholine in 50 mM KPB (pH 7.0), and 40 µL of extract. Likewise, the butyrylcholinesterase (BChE) assay consisted of 100 µL of 0.5 µg/mL equine serum BChE (≥10 units/mg protein), 50 µL of 0.4 mM butуlthioсholine (BTCh) in 50 mM KPB (pH 7.0) containing MgCl_2_ (1 mM), 10 µL of 16 mM DTNB in 50 mM KPB (pH 7.0), and 40 µL of extract. Inhibitory activity was monitored at 412 nm using the microplate reader. The inhibition percentage was then calculated as above.

Beta-secretase (BACE-1) inhibitory activity was measured using a BACE1 FRET Assay Kit (Sigma-Aldrich, St. Louis, MO, USA) with slight modifications [22]. The assay consisted of 2 µL of 0.3 U/µL BACE-1, 20 µL of 50 mM BACE-1 substrate (Rh-EVNLDAEFK-Quencher in 50 mM ammonium bicarbonate) in BACE-1 assay buffer (50 nM sodium acetate), and 20 µL of extract. The enzyme reaction was monitored at an excitation wavelength of 545 nm and an emission wavelength of 585 nm using the microplate reader. The result was calculated as percentage inhibitory activity using the equation below.

$$\%\;\mathrm{inhibition}\;=\;\left(1-\;\;\frac{B-b}{A-a}\right)\;\times \;100,$$

where *A* is the absorbance of reaction with enzyme (control), *a* is the absorbance of reaction without enzyme (control blank), *B* is the absorbance of the enzyme reaction with extract (sample), and *b* is the absorbance of the reaction with extract but without enzyme (sample blank).

High-throughput angiotensin-converting enzyme (ACEX) inhibitory activity was determined according to a previously described assay with slight modifications [30]. The reaction consisted of 3 µL of 0.5 U/mL rabbit lung ACE (≥2 unit/mg), 30 µL of 3 mM hippuryl-histidyl-leucine (HHL) in 50 mМ KРВ (pH 7.0) containing 0.025 NaOH and 3 M NaCl, and 50 µL of extract. The mixture was then incubated at 37 °C for 30 min in the dark. To stop the enzyme reaction, 177 µL of 0.28 M NaOH was added to the mixture. Then, 15 µL of 20 mg/mL *o*-phthaldialdehyde in aqueous methanol was added to visualize the enzyme reaction, and 25 µL of 3 M HCl was added to neutralize the reaction. The reaction was monitored at an excitation wavelength of 360 nm and an emission wavelength of 485 nm using the microplate reader. Results were calculated as percentage inhibitory activity, similar to the BACE-1 assay.

All enzymes, chemicals, and reagents used in the enzyme inhibitory assays were purchased from Sigma-Aldrich (St. Louis, MO, USA).

### 2.6. Statistical Analysis

The experiments were performed in triplicate (*n* = 3). The results were expressed as mean ± standard deviation (SD). Statistical analysis was performed using one-way analysis of variance (ANOVA) and a Duncan’s multiple comparison test with significant differences between values at *p* < 0.05.

## 3. Results

### 3.1. Microbial Numbers of Fermented Tea Leaves

Microbiological analysis of fermented tea leaves gave microbial numbers of total viable bacteria, lactic acid bacteria, and yeast and mold as 6.57, 6.66, and 6.49 log colony-forming units (CFU)/g sample, respectively (Table 1). Similar results were reported by Ketwal et al. (2014), suggesting that total viable bacteria and yeast and mold detected in fermented tea leaves (Cha-miang) were in the range of 6–10 log CFU/g sample [31], while lactic acid bacteria detected in Cha-miang were in the range of 6–8 CFU/g sample [6].

### 3.2. Nutritive Values

Nutritive values per 100 g dry weight (DW) suggested that fermented tea leaves possessed significantly higher energy levels (389.89 kcal) than steamed and fresh tea leaves (386.49 and 372.38 kcal, respectively) (Table 2). The high energy detected in fermented tea leaves was contributed from the high fat and carbohydrate contents. Low fat content was detected in fresh and steamed tea leaves (1.76 and 1.75 g, respectively); however, a significantly higher amount was detected in fermented tea leaves (3.20 g). High carbohydrate content was found in steamed and fermented tea leaves (67.99 and 67.69 g, respectively), while a significantly lower amount was found in fresh tea leaves (38.74 g). Fresh tea leaves contained the highest content of total dietary fiber, as well as soluble and insoluble dietary fibers (88.06, 15.23, and 72.83 g, respectively). Insoluble dietary fiber in fresh samples was approximately 4.8 times higher than soluble dietary fiber. Steamed and fermented tea leaves possessed 2.6–2.9 times higher insoluble dietary fiber than soluble dietary fiber. Fresh tea leaves also expressed a significantly higher amount of protein (50.40 g) than steamed and fermented tea leaves (24.70 and 22.58 g, respectively). Ash content was found in small amounts in all samples (5.57–9.10 g).

Major minerals found in all tea samples were calcium, sodium, potassium, and magnesium. Fresh tea leaves contained the highest amounts of calcium (587.69 mg), potassium (2726.64 mg), and magnesium (244.32 mg), while the steaming process significantly reduced quantities to 511.31 mg of calcium, 1432.62 mg of potassium, and 146.54 mg of magnesium. Fermentation further decreased calcium and magnesium contents to 453.65 mg and 127.82 mg, respectively. However, compared to the steaming process, potassium increased after fermentation (1745.96 mg). Interestingly, the highest sodium content (706.04 mg) was observed in fermented tea leaves, followed by fresh (227.15 mg) and steamed (106.98 mg) tea leaves. Iron and zinc were found in trace amounts in all samples. Fresh tea leaves contained 7.11 mg of iron and 8.65 mg of zinc. Steaming and fermenting processes reduced the contents of both minerals. Steamed tea leaves contained 0.96 mg of iron and 1.75 mg of zinc, while fermented tea leaves contained 6.72 mg of iron and 2.01 mg of zinc.

For vitamins, vitamin B1 in steamed tea leaves (4.17 mg) increased significantly compared to fresh tea leaves (2.52 mg), which possessed similar vitamin B1 content to fermented tea leaves (2.56 mg). Other vitamins, namely, vitamins B2, B3, and C, were found to be the highest in fresh tea leaves (1.45, 10.78, and 58.69 mg, respectively). Steaming and fermenting processes further reduced the contents of these vitamins, while no vitamin C was detected in fermented tea leaves.

### 3.3. Phenolic Profiles and Antioxidant Activities

Five detected flavonoids including cyanidin, myricetin, quercetin, kaempferol, and apigenin, and three detected phenolic acids including gallic acid, caffeic acid, and *p*-coumaric acid were identified in tea samples using high-performance liquid chromatography (HPLC) and the set of standards indicated in Section 2.3 (Table 3). Among flavonoids, quercetin was detected at the highest content (184.30–280.47 mg/100 g dry weight (DW)), followed by kaempferol (116.71–164.47 mg/100 g DW), cyanidin (34.22–54.41 mg/100 g DW), apigenin (31.90 mg/100 g DW), and myricetin (9.01–28.86 mg/100 g DW). All flavonoids except apigenin were detected in all tea samples, while apigenin was only detected in fermented tea leaves. Fresh and steamed tea leaves contained the two most abundant flavonoids including quercetin and kaempferol at higher contents than detected in fermented tea leaves. On the other hand, fermented tea leaves contained higher contents of cyanidin than fresh and steamed tea leaves, while myricetin content was higher in steamed and fermented tea leaves than detected in fresh tea leaves.

Among phenolic acids detected in fresh, steamed, and fermented tea leaves, gallic acid was found at the highest content (83.13–395.08 mg/100 g DW), followed by caffeic acid (3.75–9.20 mg/100 g DW) and *p*-coumaric acid (8.70–14.79 mg/100 g DW). Interestingly, gallic acid and caffeic acid were detected in all tea samples, while *p*-coumaric acid was only detected in fresh and steamed tea leaves. Fresh and steamed tea leaves contained higher amounts of all detected phenolic acids than fermented tea leaves.

The HPLC analysis indicated that the identified phenolics were found at higher quantities in fresh and steamed tea leaves; however, spectrophotometric analysis of total phenolic content (TPC) suggested otherwise (Table 3). Fermented tea leaves exhibited a TPC of 102.60 mg gallic acid equivalent (GAE)/g DW, significantly higher than steamed (97.11 mg GAE/g DW) and fresh tea leaves (25.74 mg GAE/g DW).

The TPCs concurred with the antioxidant activities determined by 2,2-diphenyl-1-picrylhydrazyl (DPPH) radical scavenging, ferric ion reducing antioxidant power (FRAP), and oxygen radical absorbance capacity (ORAC) assays (Table 4). The DPPH radical scavenging activity of fermented tea leaves (690.26 µmol Trolox equivalent (TE)/100 g DW) was significantly higher than steamed (649.91 µmol TE/100 g DW) and fresh (410.91 µmol TE/100 g DW) tea leaves. Likewise, the same trend was observed with FRAP and ORAC activities, in which fermented tea leaves exhibited significantly higher antioxidant activities (311.45 and 1539.22 µmol TE/g DW, respectively) than steamed (235.42 and 1508.49 µmol TE/g DW, respectively) and fresh (30.45 and 499.11 µmol TE/g DW, respectively) tea leaves.

### 3.4. In Vitro Enzyme Inhibitory Activities

Enzyme inhibitory assays were performed using lipase as the key enzyme to control obesity, α-amylase and α-glucosidase to test for diabetes, angiotensin-converting enzyme (ACE) to control hypertension, and acetylcholinesterase (AChE), butyrylcholinesterase (BChE), and β-secretase (BACE-1) to control Alzheimer’s disease (AD). Results for in vitro enzyme inhibitory activities were compared among fresh, steamed, and fermented tea leaves, as shown in Table 5.

Results suggested that steamed tea leaves exhibited significantly higher lipase inhibition (50.37%) than fermented (39.06%) and fresh (12.27%) tea leaves, using the extract concentration of 1 mg/mL. Fresh and steamed tea leaves exhibited higher anti-α-amylase activities (20.65–21.02% inhibition) than fermented tea leaves (15.60% inhibition) using the same extract concentration (1.25 mg/mL). However, steamed tea leaves exhibited the highest inhibition against α-glucosidase activity (84.41%), followed by fresh (72.72%) and fermented (62.04%) tea leaves, using the same extract concentration (1.25 mg/mL). On the other hand, fermented tea leaves exhibited higher ACE inhibitory activity (92.54%) than steamed (90.91%) and fresh (77.43%) tea leaves, using the extract concentration of 1.11 mg/mL. Similar results were observed with AChE and BChE inhibitory activities. Fermented tea leaves exhibited higher inhibitory activities (90.19% AChE inhibition and 66.58% BChE inhibition) than steamed (61.75% AChE inhibition and 37.12% BChE inhibition) and fresh (59.13% AChE inhibition and 17.80% BChE inhibition) tea leaves, using the extract concentration of 1 mg/mL. However, opposite results were observed with BACE-1, another key enzyme in controlling AD. Results of BACE-1 inhibition suggested that fresh tea leaves exhibited higher BACE-1 inhibitory activity (90.70%) than steamed (80.47%) and fermented (67.89%) teas, using the same extract concentration of 8 mg/mL.

## 4. Discussion

Cha-miang (*Camellia sinensis* var. assamica) is a traditional fermented tea that is locally consumed in Northern Thailand. Two major procedures are required for preparation of fermented tea, i.e., steaming and fermenting processes. Fermented tea leaves were previously reported to possess different amounts of phenolic compounds compared to fresh and steamed tea leaves [32], with nutritional biotransformation detected during the steaming and fermenting processes [6], leading to variations in health properties. However, limited information exists on the comparison of nutritive values, phenolic profiles, antioxidant activities, and health properties among fresh, steamed, and fermented tea leaves. Our results showed that (i) fermentation caused nutritional changes in tea leaves that were compatible with other nutrient-rich plants, (ii) fermentation possibly caused structural changes and varied the quantities of bioactive compounds, leading to increased antioxidant activities, and (iii) these changes also caused variations in in vitro health properties through inhibitions of key enzymes that control obesity (lipase), diabetes (α-amylase and α-glucosidase), hypertension (angiotensin-converting enzyme (ACE)), and Alzheimer’s disease (AD) (acetylcholinesterase (AChE), butyrylcholinesterase (BChE), and β-secretase (BACE-1)) compared to fresh and steamed tea leaves. This information will be useful to promote this cultural food as an alternative source of nutrients, phenols, and antioxidants with health benefits.

Nutritional biotransformation was observed during the steaming and fermenting processes. Steaming caused an increase in energy, carbohydrate, and vitamin B1, while fermentation elevated fat, sodium, and potassium contents, compared to fresh tea leaves. Similar results were previously observed, and comparisons of nutritional components in fresh and fermented tea leaves suggested an increase in fat and sugar after the fermentation process [6]. An increase in fat might be due to the lipase activity of microorganisms to break down fat compounds (such as fatty acids and glycerols) [33], thus increasing the availability of fat content in the fermented product. Furthermore, these microorganisms could digest fibers to sugars, causing the decrease in fiber content and alternatively affecting the total carbohydrate content [34,35]. The decrease in protein content might also be the result of amino-acid usage in these microorganisms [34,35]. Compared to fresh tea leaves, the decrease in most mineral contents detected in steamed and fermented tea leaves might be the result of heat treatment at 100 °C and possibly the usage of these macrominerals in enzyme activities and energy production of microorganisms [36]. However, the increase in Na and K after fermentation might be due to the digestion of covalent bonds in mineral–food matrix complexes, leading to increased bioavailability [35]. Similarly, the decrease in most vitamin contents might be due to the application of 100 °C during the steaming process and the usage of microorganisms [36]. Nutritional values per 100 g fresh weight (FW) of fermented tea leaves with 84.32% moisture content consisted of 10.63 g of carbohydrate, 3.53 g of protein, 0.50 g of fat, 1.02 g of ash, and 7.84 g of total dietary fiber (2.16 and 5.68 g of soluble and insoluble fibers, respectively). The protein content of fermented tea leaves was comparable to leaves of star gooseberry (*Phyllanthus acidus*), *Lasia spinosa*, *Cuminum cyminum*, *Tamarindus indica*, *Polygonum odoratum*, *Diplazium esculentum*, *Anethum graveolens*, and *Coccinia grandis* at 3.3–3.7 g/100 g FW [33]. The carbohydrate content of fermented tea leaves was comparable to leaves of *Ipomoea batatas*, *Senna siamea*, *Erythrina subumbrans*, and *Tamarindus indica* at 9.1–10.4 g/100 g FW [37], while the fat content of fermented tea leaves was comparable to leaves of *Lasia spinosa*, *Polygonum odoratum*, *Anethum graveolens*, *Acacia pennata* subsp. *Insuavis*, and *Spinacia oleracea* at 0.4–0.6 g/100 g FW [37]. The total dietary fiber content of fermented tea leaves was comparable to leaves of *Cuminum cyminum*, *Sesbania grandiflora*, *Piper sarmentosum*, *Marsilea crenata*, and *Leucaena leucocephala* at 5.9–7.9 g/100 g FW [37]. Moreover, these nutrients could affect the bioactivities of tea samples. For example, vitamin C could act as antioxidant; thus, antioxidant activities observed in tea samples might be a result of this vitamin (other than the biological function of phenolics). Fiber, especially soluble fiber, could also retard the absorption of sugar; thus, it helps supporting the antidiabetic property.

Interestingly, compared to fresh tea leaves, most nutrients declined after fermentation such as protein, total dietary fiber, calcium, potassium, magnesium, zinc, vitamin B2, niacin, and vitamin C. Compared to steamed tea leaves, most vitamins were also decreased after fermentation. These results were in contrast with some bioactive compounds, in which previous reports indicated that some bioactive compounds increased (i.e., (−)-epicatechin content was increased after 20 days of fermentation), while others decreased (i.e., (+)-catechin and (−)-epicatechin-3-gallate) significantly [32]. Furthermore, even though catechins are the main phenolic compounds in tea, previous studies reported that catechins were drastically reduced during tea fermentation [32,38]. Therefore, we focused on other phenolics and used many standards to identify changes during steaming and fermenting processes in an attempt to explain the bioactivities of tea leaves. Fermentation was previously reported to cause a structural change in bioactive compounds [39,40]. A previous study suggested that fermentation reduced the phenolic content as these compounds broke down or polymerized into new strands of polymers [39]. Dihydrochalcone, as one of the major flavonoids detected in tea, also noticeably decreased during fermentation due to partial oxidization [40]. Likewise, the absence of *p*-coumaric acid in fermented tea leaves in our experiment indicated biodegradation during fermentation, while detection of apigenin that was only found in fermented tea leaves also possibly resulted from structural changes of bioactive compounds during fermentation. Interestingly, an increase in phenolic acid content was observed after the steaming process. Our results concurred with a previous study, suggesting that citrus fruit (*Citrus sinensis* (L.) Osbeck) peels exhibited higher total phenolic contents (TPCs) after heat treatment at 100 °C [41]. A previous study also suggested that the high temperature weakened plant cell walls, thus releasing more phenolics into the solvent [42]. Phenolics are normally present in bound form with the food matrix [43]; thus, the high temperature was able to break down interactions between the phenolics and food matrix, leading to higher phenolic content detected in steamed tea leaves than in the fresh form. Phenolic content is often related to antioxidant activity [44]. This finding concurred with our results showing that both TPCs and antioxidant activities increased significantly after fermentation. A previous study also supported our findings that fermented tea exhibited higher antioxidant activity than fresh-brewed green tea [45].

To control obesity through limitation of fat absorption, the lipase inhibitory activities of tea samples were investigated. Our results suggested that steamed and fermented tea leaves exhibited higher lipase inhibitory activities than fresh tea leaves, correlating with a previous report indicating that fermented green tea showed in vitro inhibitory activity against lipase enzyme, with a half maximal inhibitory concentration (IC_50_) of 0.48 mg/mL and in vivo ameliorated property against postprandial plasma lipid in rats fed with fermented green tea (500 mg/kg body weight) for 2 weeks [46]. Black tea and its fermented form, kombucha (5 mL/kg body weight), were reported to reduce lipase enzyme activity in alloxan-induced diabetic rats within 30 days [47]. Interestingly, the lipase inhibitory action of these tea leaves might result from the biological functions of their bioactive compounds. A previous study revealed that quercetin, as the most abundant flavonoid detected in our tea leaves, exhibited strong inhibitory potential against lipase enzymes with an IC_50_ value of 6.1 µM [48], while the most abundant phenolic acid as gallic acid exhibited stronger lipase inhibitory activity with an IC_50_ value of 0.47 nM [49]. In addition to phenolics, short-chained peptides and free amino acids also act as lipase inhibitors [48]. Heptapeptides and amide tripeptides also effectively inhibited the pancreatic lipase reaction [50,51], while free amino acids including l-proline, l-alanine, and aspartic acid inhibited pancreatic lipase in a dose-dependent manner with IC_50_ values of 0.01, 0.14, and 0.02 µM, respectively [49]. Although fermented tea leaves possessed higher TPCs than steamed and fresh tea leaves, and the lipase inhibitory activity of steamed tea leaves was higher than that of fermented and fresh tea leaves, it is possible that the lipase inhibition observed in these tea leaves might result from the biological function of both phenolics and peptides/free amino acids degraded from protein during steaming and fermenting processes.

The control of diabetes was investigated through inhibition of the carbohydrate hydrolyzing enzymes, α-amylase and α-glucosidase. Bioactive compounds detected in tea leaves effectively inhibited these enzymes. Myricetin exhibited an IC_50_ value of 5 µM [41], while kaempferol, cyanidin, and quercetin exhibited IC_50_ values ≥500 µM against α-amylase [40]. Interestingly, these flavonoids effectively inhibited α-glucosidase, with the IC_50_ values ranging from 4 to 12 µM [42]. Gallic acid, caffeic acid, and *p*-coumaric acid exhibited the IC_50_ values of 737, 828, and >3000 µM, respectively, against α-glucosidase activities [52]. The effectiveness of these bioactive compounds led to higher α-amylase and α-glucosidase inhibitions being observed in fresh and steamed tea leaves with higher flavonoid and phenolic acid contents than fermented tea leaves.

The renin–angiotensin–aldosterone system (RAAS) hormonal cascade is one of the most significant pathways to preserve hemodynamic stability, with ACE as the key enzyme in this mechanism to control hypertension. Quercetin, kaempferol, and apigenin acted as ACE inhibitors with IC_50_ values of 43, 178, and 196 mM, respectively [53], while myricetin inhibited ACE at a similar rate to quercetin [54]. However, the IC_50_ value of caffeic acid was 430 µM [55], while gallic acid and *p*-coumaric acid exhibited 34.3% and 16.8% inhibitions, respectively, using a concentration of 4 mg/mL [56]. On the basis of this information, these phenolics were considered as weak to moderate ACE inhibitors. Interestingly, the ACE inhibitory activities of tea samples possibly resulted from other types of ACE inhibitors. ACE inhibitors have been identified in natural sources such as peptides from plants (corn, sesame, green beans, rice, and tea), animals (milk, eggs, and fish), and microorganisms [57,58], while ACE inhibitory peptides with particular amino-acid sequences were enzymatically released from protein precursors during food processing [59]. Fermented tea leaves contained lower phenolic contents than fresh and steamed tea leaves but exhibited higher ACE inhibitory activities. Thus, the high ACE inhibition observed in fermented tea leaves might result from the biological function of peptides degraded from protein during tea fermentation rather than phenolics.

Two main hypotheses of AD are proposed as the cholinergic and β-amyloid hypotheses. The former occurs from excessive cholinesterase enzymes (AChE and BChE), leading to neurotransmitter loss, while the latter leads to the formation of β-amyloid plaque by BACE-1, leading to β-amyloid aggregation [60]. A previous study suggested that quercetin was a strong AChE inhibitor with an IC_50_ value of 25.9 µM [61] but a weaker BChE inhibitor with an IC_50_ value of 177.8 µM [61]. Kaempferol and myricetin inhibited AChE with IC_50_ values of 92.8 and 37.8 µM, respectively [62], and inhibited BChE with IC_50_ values of 71.0 and 43.1 µM, respectively [62]. Caffeic acid inhibited AChE with an IC_50_ value of 23 µM and BChE with an IC_50_ value of 31 µM [63]. According to this information, fresh and steamed tea leaves should exhibit higher AChE and BChE inhibition than fermented tea leaves since they possessed higher flavonoid and phenolic acid contents. However, the results showed an opposing trend, whereby fermented tea leaves exhibited higher AChE and BChE inhibitions than fresh and steamed tea leaves. Currently, different types of peptides have been designed to act as potential AChE and BChE inhibitors, as a new approach to overcome AD occurrence [64,65,66]. Interestingly, some of these peptides are derived from the AD synthetic drug, galantamine [65], as well as from protein hydrolysates from natural sources such as hemp seed [66]. Other than peptides, a complex of polysaccharide–peptide conjugates was also reported to act as an AChE and BChE inhibitor [67]. Thus, high AChE and BChE inhibition in fermented tea leaves might be the result of peptides degraded from protein during tea fermentation. Results of BACE-1 inhibition also suggested that fresh tea leaves exhibited higher BACE-1 inhibitory activity than steamed and fermented tea leaves. Previous research suggested that food rich in flavonoids such as green tea, blueberry, and cocoa inhibited BACE-1 or disrupted amyloid β-aggregation [68]. A previous study suggested that quercetin, kaempferol, myricetin, and apigenin inhibited BACE-1 activities with IC_50_ values of 5.4, 14.7, 2.8, and 38.5 µM, respectively [69]. Thus, quercetin and myricetin were considered as strong BACE-1 inhibitors. Moreover, *p*-coumaric acid inhibited BACE-1 in a noncompetitive manner with an IC_50_ value of 0.9 µM [70]. No report on the BACE-1 inhibitory activity of caffeic acid is currently available; however, caffeic acid was proven capable of improving spatial cognition and memory unit in Aβ_25–35_-injected AD mice [71]. Gallic acid also reduced Aβ_1–42_ aggregation and neurotoxicity in an APP/PS1 transgenic mouse model [72]. According to this information, higher BACE-1 inhibitory activities observed in fresh and steamed tea leaves than in fermented tea leaves resulted from the greater flavonoid and phenolic acid contents in nonfermented tea leaves.

The information received from this study would be useful for the promotion of Cha-miang consumption as a historical and unique product of northern Thailand with many health benefits. This information also suggests that Cha-miang could be used as a source of nutrients and bioactive compounds to develop other functional foods and drinks. Moreover, the knowledge gained from this research would support local tea fermentation as an alternative method of cultural food preservation, resulting in a fermented product with unique favor and taste, and leading to the sustainable conservation of local lifestyle.

## Figures and Tables

**Table 1:** Microbial numbers of viable bacteria, lactic acid bacteria, and yeast and mold in fermented tea (log colony-forming units (CFU)/g sample).

Sample	Microbial Numbers (log CFU/g Sample)		
	Total Viable Bacteria	Lactic Acid Bacteria	Yeast and Mold
Fermented tea	6.57 ± 0.07	6.66 ± 0.07	6.49 ± 0.01

All data are shown as the mean ± standard deviation (SD) of triplicate experiments (*n* = 3).

**Table 2:** Nutritional compositions of fresh, steamed, and fermented tea leaves.

Nutrients	Nutritive Values (per 100 g Dry Weight)		
	Fresh Tea Leaves	Steamed Tea Leaves	Fermented Tea Leaves
**Energy (kcal)**	372.38 ± 1.32 ^c^	386.49 ± 0.67 ^b^	389.89 ± 0.73 ^a^
**Protein (g)**	50.40 ± 1.08 ^a^	24.70 ± 1.32 ^b^	22.58 ± 1.44 ^b^
**Fat (g)**	1.76 ± 0.04 ^b^	1.75 ± 0.13 ^b^	3.20 ± 0.23 ^a^
**Carbohydrate (g)**	38.74 ± 1.33 ^b^	67.99 ± 1.78 ^a^	67.69 ± 2.14 ^a^
**Total dietary fiber (g)**	88.06 ± 4.24 ^a^	51.99 ± 3.22 ^b^	50.11 ± 3.56 ^b^
Soluble dietary fiber (g)	15.23 ± 0.88 ^a^	13.48 ± 0.91 ^a^	13.82 ± 1.67 ^a^
Insoluble dietary fiber (g)	72.83 ± 3.35 ^a^	38.52 ± 2.31 ^b^	36.28 ± 1.89 ^b^
**Ash (g)**	9.10 ± 0.29 ^a^	5.57 ± 0.33 ^c^	6.53 ± 0.47 ^b^
**Minerals**			
Calcium (mg)	587.69 ± 14.86 ^a^	511.31 ± 32.49 ^b^	453.65 ± 31.60 ^c^
Sodium (mg)	227.15 ± 15.31 ^b^	106.98 ± 2.67 ^c^	706.04 ± 54.82 ^a^
Potassium (mg)	2726.64 ± 82.13 ^a^	1432.62 ± 87.39 ^c^	1745.96 ± 172.99 ^b^
Magnesium (mg)	244.32 ± 13.33 ^a^	146.54 ± 7.65 ^b^	127.82 ± 5.53 ^b^
Iron (mg)	7.11 ± 0.07 ^a^	5.96 ± 0.35 ^b^	6.72 ± 0.42 ^a^
Zinc (mg)	8.65 ± 0.43 ^a^	1.75 ± 0.03 ^b^	2.01 ± 0.11 ^b^
**Vitamin** **s**			
Vitamin B1 (mg)	2.52 ± 0.13 ^b^	4.17 ± 0.42 ^a^	2.56 ± 0.18 ^b^
Vitamin B2 (mg)	1.45 ± 0.11 ^a^	0.67 ± 0.06 ^b^	0.32 ± 0.02 ^c^
Niacin (mg)	10.78 ± 0.17 ^a^	3.70 ± 0.32 ^b^	2.69 ± 0.19 ^c^
Vitamin C (mg)	58.69 ± 3.56 ^a^	46.08 ± 4.00 ^b^	ND

All data are shown as the mean ± standard deviation (SD) of triplicate experiments (*n* = 3). ND: not detected. The letters indicate significant differences (*p* < 0.05) of the same nutrients in different tea samples using one-way analysis of variance (ANOVA) and Duncan’s multiple comparison test.

**Table 3:** Identification of flavonoids and phenolic acids in fresh, steamed, and fermented tea leaves.

Phenolics (mg/100 g DW)	Tea Leaves		
	Fresh	Steamed	Fermented
**Flavonoids**			
Cyanidin	44.54 ± 3.15 ^b^	34.22 ± 0.45 ^c^	54.41 ± 3.65 ^a^
Myricetin	9.01 ± 0.06 ^b^	25.48 ± 2.24 ^a^	28.86 ± 2.51 ^a^
Quercetin	280.47 ± 5.22 ^a^	247.91 ± 18.09 ^b^	184.30 ± 12.25 ^c^
Kaempferol	164.47 ± 4.79 ^a^	156.56 ± 9.12 ^a^	116.71 ± 9.77 ^b^
Apigenin	ND	ND	31.90 ± 2.68
**Phenolic acids**			
Gallic acid	237.18 ± 17.24 ^b^	395.08 ± 21.08 ^a^	83.13 ± 5.73 ^c^
Caffeic acid	9.20 ± 0.56 ^a^	7.86 ± 0.80 ^b^	3.75 ± 0.32 ^c^
*p*-Coumaric acid	14.79 ± 0.60 ^a^	8.70 ± 0.12 ^b^	ND
**TPCs (mg GAE/g DW)**	25.74 ± 1.21 ^c^	97.11 ± 2.83 ^b^	102.60 ± 1.40 ^a^

All data are shown as the mean ± standard deviation (SD) of triplicate experiments (*n* = 3). DW: dry weight; GAE: gallic acid equivalent; ND: not detected. The letters indicate significant differences (*p* < 0.05) of the same phenolics in different tea samples using one-way analysis of variance (ANOVA) and Duncan’s multiple comparison test.

**Table 4:** Total phenolic content (TPC) and antioxidant activities detected by 2,2-diphenyl-1-picrylhydrazyl (DPPH) radical scavenging, ferric ion reducing antioxidant power (FRAP), and oxygen radical absorbance capacity (ORAC) assays of fresh, steamed, and fermented teas.

Tea Leaves	Antioxidant Activities		
	DPPH Radical Scavenging Assay (µmol TE/100 g DW)	FRAP Assay (µmol TE/g DW)	ORAC Assay (µmol TE/g DW)
Fresh	410.91 ± 9.62 ^c^	30.45 ± 2.62 ^c^	499.11 ± 9.91 ^c^
Steamed	649.91 ± 7.07 ^b^	235.42 ± 5.60 ^b^	1508.49 ± 85.52 ^b^
Fermented	690.26 ± 10.66 ^a^	311.45 ± 9.38 ^a^	1539.22 ± 83.55 ^a^

All data are shown as the mean ± standard deviation (SD) of triplicate experiments (*n* = 3). DW: Dry weight; TE: Trolox equivalent. The letters indicate significant differences (*p* < 0.05) of different tea samples in the same antioxidant assay using one-way analysis of variance (ANOVA) and Duncan’s multiple comparison test.

**Table 5:** In vitro enzyme inhibitory activities against lipase, α-amylase, α-glucosidase, angiotensin-converting enzyme (ACE), acetylcholinesterase (AChE), butyrylcholinesterase (BChE), and β-secretase (BACE-1) in fresh, steamed, and fermented tea leaves.

Tea Leaves	% Inhibition						
	^1^ Lipase	^2^ α-Amylase	^2^ α-Glucosidase	^3^ ACE	^1^ AChE	^1^ BChE	^4^ BACE-1
Fresh	12.27 ± 0.92 ^c^	20.65 ± 1.95 ^a^	72.72 ± 1.17 ^b^	77.43 ± 2.61 ^c^	59.13 ± 1.03 ^c^	17.80 ± 1.22 ^c^	90.70 ± 0.49 ^a^
Steamed	50.37 ± 3.12 ^a^	21.02 ± 1.16 ^a^	84.41 ± 3.31 ^a^	90.91 ± 0.68 ^b^	61.75 ± 2.34 ^b^	37.12 ± 2.12 ^b^	80.47 ± 0.99 ^b^
Fermented	39.06 ± 0.21 ^b^	15.60 ± 1.30 ^b^	62.04 ± 2.73 ^c^	92.54 ± 2.70 ^a^	90.19 ± 2.84 ^a^	66.58 ± 6.52 ^a^	67.89 ± 4.26 ^c^

All data are shown as the mean ± standard deviation (SD) of triplicate experiments (*n* = 3). The letters indicate significant differences (*p* < 0.05) of different samples in the same enzyme assay using one-way analysis of variance (ANOVA) and Duncan’s multiple comparison test. ^1^ Final concentration = 1.00 mg/mL; ^2^ final concentration = 1.25 mg/mL; ^3^ final concentration = 1.11 mg/mL; ^4^ final concentration = 8.00 mg/mL.

## Data Availability

Data is contained within this article and supplementary material.

## Supplementary Materials

The following are available online at https://www.mdpi.com/2304-8158/10/1/117/s1: Table S1. Nutrient compositions of fresh, steamed, and fermented tea leaves per 100 g fresh weight; Table S2. The validation parameters used for HPLC analysis; Figure S1. High-performance liquid chromatograms of fresh, steamed, and fermented tea leaves detected at 280, 325, and 524 nm.

## References

[1] Pripdeevech P., Machan T. (2011). Fingerprint of volatile flavour constituents and antioxidant activities of teas from Thailand. Food Chem..

[2] Khanongnuch C., Unban K., Kanpiengjai A., Seanjum C. (2017). Recent research advances and ethno-botanical history of miang, a traditional fermented tea (*Camellia sinensis* var. *assamica*) of Northern Thailand. J. Ethn. Foods..

[3] Kim Y., Goodner L.K., Park D.J., Choi J., Talcott T.S. (2011). Changes in antioxidant phytochemicals and volatile composition of *Camellia sinensis* by oxidation during tea fermentation. Food Chem..

[4] Tanasupawat S., Pakdeeto A., Thawai C., Yukphan P., Okada S. (2007). Identification of lactic acid bacteria from fermented tea leaves (*miang*) in Thailand and proposals of *Lactobacillus thailandensis* sp. nov., *Lactobacillus camelliae* sp. nov., and *Pediococcus siamensis* sp. nov. J. Gen. Appl. Microbiol..

[5] Phromrukachat S., Tiengburanatum N., Meechui J. (2010). Assessment of active ingredients in pickled tea. Asian J. Food Agro-Ind..

[6] Unban K., Khatthongngam N., Shetty K., Khanongnuch C. (2019). Nutritional biotransformation in traditional fermented tea (Miang) from north Thailand and its impact on antioxidant and antimicrobial activities. J. Food Sci. Technol..

[7] Aekplakorn W., Mo-suwan L. (2009). Prevalence of obesity in Thailand: A review. Obes. Rev..

[8] Aekplakorn W., Klafter A.J., Premgamone A., Dhanmun B., Chaikittiporn C., Chongsuvatwong V., Suwanprapisa T., Chaipornsupaisan W., Tiptaradol S., Lim S.S. (2007). Prevalence and management of diabetes and associated risk factors by regions of Thailand: Third National Health Examination Survey 2004. Diabetes Care.

[9] Van de Laar F.A. (2008). Alpha-glucosidase inhibitors in the early treatment of type 2 diabetes. Vasc. Health Risk Manag..

[10] De la garza A.L., Etexeberria U., Lostao P.M., Roman S.B., Barrenetxe J., Martinez A.J., Milagro F.I. (2013). Helichrysum and grapefruit extracts inhibit carbohydrate digestion and absorption, improving postprandial glucose levels and hyperinsulinemia in rats. J. Agric. Food Chem..

[11] Balasuriya B.N., Rupasinghe V.H. (2011). Plant flavonoids as angiotensin converting enzyme inhibitors in regulation of hypertension. Funct. Food Health Dis..

[12] Parihar M., Hemnani T. (2004). Alzheimer’s disease pathogenesis and therapeutic interventions. J. Clin. Neurosci..

[13] Rao A.A., Sridhar G.R., Das N.U. (2007). Elevated butyrylcholinesterase and acetylcholinesterase may predict the development of type 2 Diabetes Mellitus and Alzheimer’s disease. Med. Hypotheses.

[14] Pákáski M., Hugyecz M., Sántha P., Jancsó G., Bjelik A., Domokos Á. (2009). Capsaicin promotes the amyloidogenic route of brain amyloid precursor protein processing. Neurochem. Int..

[15] Huovila A.P., Turner J.A., Pelto-Huikko M., Kärkkäinen I., Ortiz M.R. (2005). Shedding light on ADAM metalloproteinases. Trends Biochem. Sci..

[16] Latimer G.W. (2019). Official Method of Analysis of AOAC International.

[17] Puwastien P., Siong T.E., Kantasubrata J., Craven G., Feliciano R.R., Judprasong K. (2011). ASEAN Manual of Food Analysis.

[18] Wehling R.L., Wetzel D.L. (1984). Simultaneous determination of pyridoxine, riboflavin, and thiamin in fortified cereal products by high-performance liquid chromatography. J. Agric. Food Chem..

[19] Wimalasiri P., Wills R.B.H. (1985). Simultaneous analysis of thiamin and riboflavin in foods by high-performance liquid chromatography. J. Chromatogr..

[20] Windahl K.L., Trenerry V.C., Ward C.M. (1998). The determination of niacin in selected foods by capillary electrophoresis and high performance liquid chromatography: Acid extraction. Food Chem..

[21] Ward C.M., Trenerry V.C. (1997). The determination of niacin in cereals, meat and selected foods by capillary electrophoresis and high performance liquid chromatography. Food Chem..

[22] Sritalahareuthai V., Temviriyanukul P., On-nom N., Charoenkiatkul S., Suttisansanee U. (2020). Phenolic profiles, antioxidant, and inhibitory activities of *Kadsura heteroclita* (Roxb.) Craib and *Kadsura coccinea* (Lem.) A.C. Sm. Foods.

[23] Ainsworth E.A., Gillespie K.M. (2007). Estimation of total phenolic content and other oxidation substrates in plant tissues using Folin–Ciocalteu reagent. Nat. Protoc..

[24] Sripum C., Kukreja R.K., Charoenkiatkul S., Kriengsinyos W., Suttisansanee U. (2017). The effect of extraction conditions on antioxidant activities and total phenolic contents of different processed Thai jasmine rice. Int. Food Res. J..

[25] Fukumoto L., Mazza G. (2000). Assessing antioxidant and prooxidant activities of phenolic compounds. J. Agric. Food Chem..

[26] Benzie I.F.F., Strain J.J. (1996). The ferric reduction ability of plasma (FRAP) as a measure of antioxidant power: The FRAP Assay. Anal. Biochem..

[27] Ou B., Huang D., Hampsch-Woodili M.H., Flanagan J.A., Deemer E.K. (2002). Analysis of antioxidant activities of common vegetables employing oxygen radical absorbance capacity (ORAC) and ferric reducing power (FRAP) assays: A comparative study. J. Agric. Food Chem..

[28] Hinkaew J., Sahasakul Y., Tangsuphoom N., Suttisansanee U. (2020). The effect of cultivar variation on total phenolic contents and antioxidant activities of date palm fruit (*Phoenix dactylifera* L.). Curr. Res. Nutr. Food Sci..

[29] Pongkunakorn T., Watcharachaisoponsiri T., Chupeerach C., On-Nom N., Suttisansanee U. (2017). Inhibitions of key enzymes relevant to obesity and diabetes of Thai local mushroom extracts. Curr. Appl. Sci. Technol. J..

[30] Schwager L.S., Carmona K.A., Sturrock D.S. (2006). A high-throughput fluorimetric assay for angiotensin I-converting enzyme. Nat. Protoc..

[31] Ketwal S., Chueamchaitrakun P., Theppakorn T., Wongsakul S. Contents of total polyphenol, microorganisms and antioxidant capacities of pickled tea (miang) commercially available in Chiang Rai, Thailand. Proceedings of the 16th Food Innovation Asia Conference.

[32] Bouphun T., Wei X., Dan W., Zhao R., Qu Z. (2018). Dynamic changes in chemical constituents during processing of Miang (Thai fermented tea leaf) in various degree of tea leaf maturity. Int. J. Food Eng..

[33] Odunfa S.A. (1983). Biochemical changes during production of ogiri, a fermented melon (*Citrullus vulgaris* Schrad) product. Plant Foods Hum. Nutr..

[34] Osman M.A. (2011). Effect of traditional fermentation process on the nutrient and antinutrient contents of pearl millet during preparation of Lohoh. J. Saudi Soc. Agric. Sci..

[35] Pranoto Y., Anggrahini S., Efendi Z. (2013). Effect of natural and *Lactobacillus plantarum* fermentation on in vitro protein and starch digestibilities of sorghum flours. Food Biosci..

[36] Karimian-Khosroshahi N., Hosseini H., Rezaei M., Khaksar R., Mahmoudzadeh M. (2016). Effect of different cooking methods on minerals, vitamins, and nutritional quality indices of rainbow trout (*Oncorhynchus mykiss*). Int. J. Food Prop..

[37] Judprasong K., Puwastien P., Rojroongwasinkul N., Nitithamyong A., Sridonpai P., Somjai A. (2015). Thai Food Composition Database.

[38] Tu Y.Y., Xia H.L., Watanabe N. (2005). The changes of catechins during the fermentation of green tea. Prikl. Biokhim. Mikrobiol..

[39] Almajano P.M., Carbó R., Jiménez L.A., Gordon H.M. (2008). Antioxidant and antimicrobial activities of tea Infusions. Food Chem..

[40] Joubert E. (1996). HPLC quantification of the dihydrochalcones, aspalathin and nothofagin in rooibos tea (*Aspalathus linearis*) as affected by processing. Food Chem..

[41] Chen L.M., Yang J.D., Liu C.S. (2011). Effects of drying temperature on the flavonoid, phenolic acid and antioxidative capacities of the methanol extract of citrus fruit (*Citrus sinensis* (L.) Osbeck) Peels. Int. J. Food Sci. Technol..

[42] Shi J., Yu J., Pohorly J., Young J.C., Bryan M., Wu Y. (2003). Optimization of the extraction of polyphenols from grape seed meal by aqueous ethanol solution. J. Food Agric. Environ..

[43] Jeong S.M., Kim S.Y., Kim D.R., Jo S.C., Nam K.C., Ahn D.U., Lee S.C. (2004). Effect of heat treatment on the antioxidant activity of extracts from citrus peels. J. Agric. Food Chem..

[44] Lee L.S., Kim S.H., Kim Y.B., Kim Y.C. (2014). Quantitative analysis of major constituents in green tea with different plucking periods and their antioxidant activity. Molecules.

[45] Somsong P., Tiyayon P., Srichamnong W. (2018). Antioxidant of green tea and pickle tea product, miang, from Northern Thailand. Acta. Hortic..

[46] Seo D.B., Jeong H.W., Kim Y.J., Kim S.K., Kim J.K., Lee J.H., Joo K.M., Choi J.K., Shin S.S., Lee S.J. (2017). Fermented green tea extract exhibits hypolipidaemic effects through the inhibition of pancreatic lipase and promotion of energy expenditure. Br. J. Nutr..

[47] Aloulou A., Hamden K., Elloumi D., Ali M.B., Hargafi K., Jaouadi B., Ayadi F., Elfeki A., Ammar E. (2012). Hypoglycemic and antilipidemic properties of kombucha tea in alloxan-induced diabetic rats. BMC Complementary Altern. Med..

[48] Martinez-Gonzalez A.I., Alvarez-Parrilla E., Díaz-Sánchez A.G., De la Rosa L.A., Núñez-Gastélum J.A., Vazquez-Flores A.A., Gonzalez-Aguilar G.A. (2017). In vitro inhibition of pancreatic lipase by polyphenols: A kinetic, fluorescence spectroscopy and molecular docking study. Food Technol. Biotechnol..

[49] De Pradhan I., Dutta M., Choudhury K., Bratati D. (2017). Metabolic diversity and in vitro pancreatic lipase inhibition activity of some varieties of *Mangifera indica* L. fruits. Int. J. Food Prop..

[50] Lunder M., Bratkovič T., Kreft S., Štrukelj B. (2005). Peptide inhibitor of pancreatic lipase selected by phage display using different elution strategies. J. Lipid Res..

[51] Stefanucci A., Dimmito P.M., Zengin G., Luisi G., Mirzaie S., Novellino E., Mollica A. (2019). Discovery of novel amide tripeptides as pancreatic lipase inhibitors by virtual screening. New J. Chem..

[52] Shobana S., Sreerama Y., Malleshi N. (2009). Composition and enzyme inhibitory properties of finger millet (*Eleusine coracana* L.) seed coat phenolics: Mode of inhibition of α-glucosidase and pancreatic amylase. Food Chem..

[53] Yang B., Liu P. (2012). Composition and health effects of phenolic compounds in hawthorn (*Crataegus* spp.) of different origins. J. Sci. Food Agric..

[54] Chen S.Y., Chu C.C., Jiang C.L., Duh P.D. (2019). The vasodilating effect and angiotensin converting enzyme inhibition activity of three dietary flavonols: Comparsion between myricetin, quercetin and morin, in vitro. J. Food Nutr. Res..

[55] Bhullar K.S., Lassalle-Claux G., Touaibia M., Rupasinghe H.V. (2014). Antihypertensive effect of caffeic acid and its analogs through dual renin–angiotensin–aldosterone system inhibition. Eur. J. Pharmacol..

[56] Nwaji N.N., Ojo O.O., Ayinla Z.A., Ajayi-Smith A.F., Mgbenka U.R., Nkwor A.N., Onuoha M.O. (2016). Antioxidant and angiotensin converting enzyme inhibition activity of *Landophia owariensis*. J. Pharmacogn. Phytochem..

[57] Iwaniak A., Minkiewicz P., Darewicz M. (2014). Food-originating ACE inhibitors, including antihypertensive peptides, as preventive food components in blood pressure reduction. Compr. Rev. Food Sci. Food Saf..

[58] Persson I.A.L., Josefsson M., Persson K., Andersson R.G. (2006). Tea flavanols inhibit angiotensin-converting enzyme activity and increase nitric oxide production in human endothelial cells. J. Pharm. Pharmacol..

[59] De Leo F., Panarese S., Gallerani R., Ceci L.R. (2009). Angiotensin converting enzyme (ACE) inhibitory peptides: Production and implementation of functional food. Curr. Pharm. Design..

[60] Hardy J., Selkoe D.J. (2002). The amyloid hypothesis of Alzheimer’s disease: Progress and problems on the road to therapeutics. Science.

[61] Min S.W., Ryu S.N., Kim D.H. (2010). Anti-inflammatory effects of black rice, cyanidin-3-O-*β*-D-glycoside, and its metabolites, cyanidin and protocatechuic acid. Int. Immunopharmacol..

[62] Katalinić M., Rusak G., Barović J.D., Šinko G., Jelić D., Antolović R., Kovarik Z. (2010). Structural aspects of flavonoids as inhibitors of human butyrylcholinesterase. Eur. J. Med. Chem..

[63] Oboh G., Agunloye O.M., Akinyemi A.J., Ademiluyi A.O., Adefegha S.A. (2013). Comparative study on the inhibitory effect of caffeic and chlorogenic acids on key enzymes linked to Alzheimer’s disease and some pro-oxidant induced oxidative stress in rats’ brain-in vitro. Neurochem. Res..

[64] Mondal P., Gupta V., Das G., Pradhan K., Khan J., Gharai P.K., Ghosh S. (2018). Peptide-based acetylcholinesterase inhibitor crosses the blood-brain barrier and promotes neuroprotection. ACS Chem. Neurosci..

[65] Yaneva S.A., Stoykova I.I., Ilieva L.I., Vezenkov L.T., Marinkova D.A., Yotova L.K., Raykova R.N., Danalev D.L. (2017). Acetylcholinesterase inhibition activity of peptide analogues of galanthamine with potential application for treatment of Alzheimers disease. Bulg. Chem. Commun..

[66] Malomo S.A., Aluko R.E. (2019). Kinetics of acetylcholinesterase inhibition by hemp seed protein-derived peptides. J. Food Biochem..

[67] Thabthimsuk S., Panya N., Owatworakit A., Choksawangkarn W. Acetylcholinesterase inhibition and antioxidant activities of polysaccharide-peptide complexes from edible mushrooms. Proceedings of the 6th International Conference on BMB: Biochemitry and Molecular Biology.

[68] Anand P., Singh B. (2013). Flavonoids as lead compounds modulating the enzyme targets in Alzheimer’s disease. Med. Chem. Res..

[69] Shimmyo Y., Kihara T., Akaike A., Niidome T., Sugimoto H. (2008). Flavonols and flavones as BACE-1 inhibitors: Structure–activity relationship in cell-free, cell-based and in silico studies reveal novel pharmacophore features. Biochim. Biophys. Acta. Gen. Subj..

[70] Youn K., Jun M. (2012). Inhibitory effects of key compounds isolated from corni fructus on BACE1 activity. Phytother. Res..

[71] Kim S., Sato Y., Mohan P.S., Peterhoff C., Pensalfini A., Rigoglioso A., Jiang Y., Nixon R.A. (2016). Evidence that the rab5 effector APPL1 mediates APP-βCTF-induced dysfunction of endosomes in down syndrome and Alzheimer’s disease. Mol. Psychiatr..

[72] Yu Y., Jans D.C., Winblad B., Tjernberg L.O., Schedin-Weiss S. (2018). Neuronal Aβ42 is enriched in small vesicles at the presynaptic side of synapses. Life Sci. Alliance.

