# Supplementary material for: The Effect of Steaming and Fermentation on Nutritive Values, Antioxidant Activities, and Inhibitory Properties of Tea Leaves
Source: Foods. 2021 Jan 8;10(1):117. doi: 10.3390/foods10010117 (PMC7827290; doi:10.3390/foods10010117)
Supplement: Supplementary file 1 [file foods-10-00117-s001.pdf]

Supplementary materials

The effect of steaming and fermentation on nutritive values, antioxidant activities, and inhibitory properties of tea leaves

Chaowanee Chupeerach[1,2], Amornrat Aursalung[1], Thareerat Watcharachaisoponsiri[1], Kanyawee Whanmek[1], Parunya Thiyajai[1], Kachakot Yosphan[1], Varittha Sritalahareuthai[1], Yuraporn Sahasakul[1,2], Chalat Santivarangkna[1,2] and Uthaiwan Suttisansanee[1,2,*]

1. Institute of Nutrition, Mahidol University, Salaya, Phuttamonthon, Nakhon Pathom 73170, Thailand; chaowanee.chu@mahidol.ac.th (C.C.); amornrat.aur@mahidol.ac.th (A.A.); thareerat.wat@hotmail.com (T.W.); kanyaweebiosci@gmail.com (K.W.); parunya.thy@mahidol.ac.th (P.T.); kachakot.yp@gmail.com (K.Y.); varittha.sri@hotmail.com (V.S.); yuraporn.sah@mahidol.ac.th (Y.S.); chalat.san@mahidol.ac.th (C.S.); uthaiwan.sut@mahidol.ac.th (U.S.)
2. Food and Nutrition Academic and Research Cluster, Institute of Nutrition, Mahidol University, Salaya, Phuttamonthon, Nakhon Pathom 73170, Thailand

* Correspondence: uthaiwan.sut@mahidol.ac.th; Tel.: +662-800-2380 ext. 422

Supplementary Table S1:

Nutrient compositions of fresh, steamed, and fermented tea leaves per 100 g fresh weight.

Nutrients	Nutritive values (per 100 g fresh weight)		
	Fresh leaves	Steamed leaves	Fermented leaves
**Energy (kcal)**	24.36 ± 0.64 [c]	78.66 ± 4.74 [a]	61.14 ± 4.52 [b]
**Moisture content (g)**	93.46 ± 0.15 [a]	79.65 ± 1.19 [c]	84.32 ± 1.13 [b]
**Protein (g)**	3.30 ± 0.00 [c]	5.02 ± 0.02 [a]	3.53 ± 0.03 [b]
**Fat (g)**	0.12 ± 0.01 [c]	0.36 ± 0.01 [b]	0.50 ± 0.00 [a]
**Carbohydrate (g)**	2.54 ± 0.14 [c]	13.85 ± 1.17 [a]	10.63 ± 1.10 [b]
**Total dietary fiber (g)**	5.76 ± 0.15 [c]	10.56 ± 0.04 [a]	7.84 ± 0.01 [b]
Soluble dietary fiber (g)	1.00 ± 0.04 [c]	2.74 ± 0.02 [a]	2.16 ± 0.11 [b]
Insoluble dietary fiber (g)	4.76 ± 0.11 [c]	7.82 ± 0.01 [a]	5.68 ± 0.12 [b]
**Ash (g)**	0.60 ± 0.01 [c]	1.13 ± 0.00 [a]	1.02 ± 0.00 [b]
**Minerals**			
Calcium (mg)	38.42 ± 0.09 [c]	103.80 ± 0.52 [a]	70.90 ± 0.19 [b]
Sodium (mg)	14.84 ± 0.66 [c]	21.75 ± 0.73 [b]	110.30 ± 0.59 [a]
Potassium (mg)	178.24 ± 1.28 [c]	290.85 ± 0.71 [a]	272.47 ± 7.33 [b]
Magnesium (mg)	15.97 ± 0.50 [c]	29.76 ± 0.19 [a]	20.00 ± 0.58 [b]
Iron (mg)	0.47 ± 0.02 [c]	1.21 ± 0.00 [a]	1.05 ± 0.01 [b]
Zinc (mg)	0.57 ± 0.02 [a]	0.36 ± 0.02 [b]	0.32 ± 0.01 [c]
**Vitamins**			
Vitamin B1 (mg)	0.17 ± 0.01 [c]	0.85 ± 0.04 [a]	0.40 ± 0.00 [b]
Vitamin B2 (mg)	0.10 ± 0.01 [b]	0.14 ± 0.01 [a]	0.05 ± 0.00 [c]
Niacin (B3) (mg)	0.71 ± 0.01 [b]	0.75 ± 0.02 [a]	0.42 ± 0.00 [c]
Vitamin C (mg)	3.84 ± 0.15 [b]	9.35 ± 0.27 [a]	ND

All data were expressed as mean ± SD of triplicate experiments; ND: not detected. [a-c] showed significant difference ($p < 0.05$) of the same nutrients in different samples using one way ANOVA followed by Duncan's *post hoc* test.

Supplementary Table S2:

The validation parameters used for HPLC analysis.

Standards	Retention time (min)	Linear range (µg/mL)	Linear regression equation	Correlation coefficient (R^2)	LOQ (µg/mL)	LOQ (µg/mL)	RSD (%)
**Phenolic acids**							
Gallic acid	5.01-5.27	0.78-200.00	y = 38.814x + 43.836	0.9998	2.66	8.07	1.37
4-Hydroxybenzoic acid	13.58-14.53	0.39-100.00	y = 54.999x - 11.469	0.9994	0.32	0.98	1.86
Chlorogenic acid	13.94-14.56	0.39-100.00	y = 45.107x - 8.1659	1.0000	0.03	0.10	1.59
Caffeic acid	17.06-17.80	0.39-100.00	y = 76.614x + 15.797	0.9998	0.59	1.78	1.70
Syringic acid	17.95-18.83	0.65-166.67	y = 37.286x - 24.557	0.9990	1.12	3.41	1.63
p-Coumaric acid	24.72-26.21	0.56-142.86	y = 44.809x - 22.611	0.9991	0.57	1.73	1.97
Ferulic acid	27.88-28.92	0.56-142.86	y = 42.477x - 16.212	0.9990	0.49	1.48	1.58
Sinapic acid	28.61-29.45	0.39-100.00	y = 63.807x + 10.841	1.0000	0.22	0.68	1.01
**Flavonoids**							
Hesperidin	38.06-38.64	0.65-333.33	y = 24.424x + 76.057	0.9990	2.56	7.77	0.64
Myricetin	39.45-40.02	0.65-166.67	y = 43.048x + 16.879	0.9999	0.37	1.12	0.32
Quercetin	44.58-44.75	0.65-166.67	y = 46.975x + 38.753	0.9999	0.19	0.58	0.10
Luteolin	44.87-45.54	0.65-166.67	y = 44.717x + 28.899	0.9999	1.19	3.62	0.52
Naringenin	45-41-45.87	0.65-333.33	y = 30.205x + 46.292	0.9999	0.46	1.38	0.45
Kaempferol	47.74-47.96	0.65-166.67	y = 47.952x + 43.855	0.9999	0.14	0.42	0.12
Apigenin	47.61-48.10	0.39-200.00	y = 40.778x + 34.994	0.9999	2.22	6.73	0.44
Isorhamnetin	48.15-48.69	1.30-333.33	y = 16.302x + 46.483	0.9992	0.45	1.37	0.39
**Anthocyanidins**							
Delphinidin	9.44-9.48	2.58-330.00	y = 1.6280x + 5.0454	0.9992	0.77	2.34	0.45
Cyanidin	16.74-16.85	2.58-330.00	y = 1.5641x + 4.4497	0.9995	0.05	0.15	0.58
Petunidin	18.26-18.33	3.91-125.00	y = 1.2904x + 4.4487	0.9951	0.78	2.36	0.28
Peonidin	36.62-36.96	2.58-330.00	y = 1.8154x + 4.1796	0.9995	3.08	9.34	0.73
Malvidin	42.51-42.61	3.91-125.00	y = 0.6093x + 4.1012	0.9945	1.82	5.53	0.24

Supplementary Figure S1:

High-performance liquid chromatograms of fresh, steamed, and fermented tea leaves detected at 280, 325, and 524 nm.

	Detected wavelength (nm)	Chromatograms
Fresh tea leaves	524	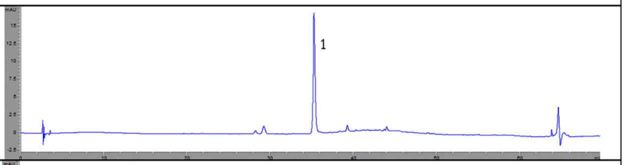
	280	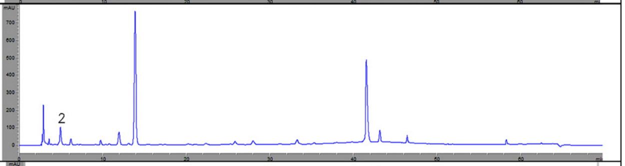
	325	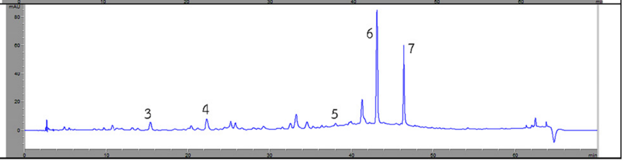
Steamed tea leaves	524	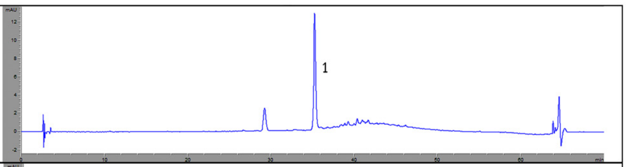
	280	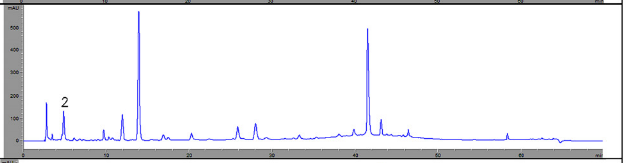
	325	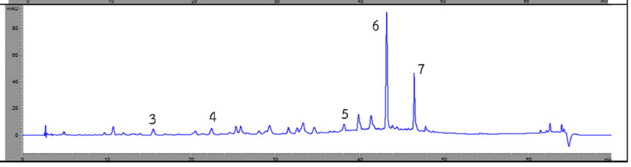
Fermented tea leaves	524	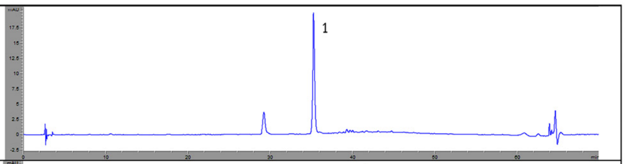
	280	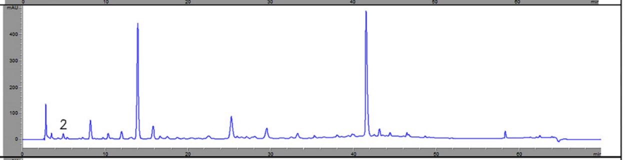
	325	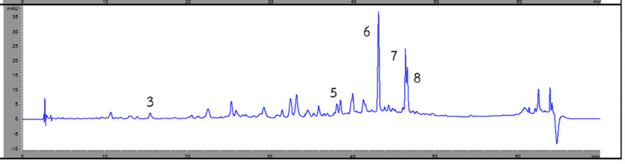

1 = cyanidin, 2 = gallic acid, 3 = caffeic acid, 4 = *p*-courmaric acid, 5 = myricetin, 6= querceitn, 7 = kaempferol, 8 = apigenin
